# Health Help-Seeking Behavior: The Scavengers’ Perspective

**DOI:** 10.3390/ijerph19116457

**Published:** 2022-05-26

**Authors:** Beenish Malik, Novel Lyndon, Yew Wong Chin, Basharat Hussain, Sehrish Malik

**Affiliations:** 1 Department of Sociology, School of Social Sciences and Humanities, University of Management and Technology, C-II Block Johar Town, Lahore 54770, Pakistan; basharat.hussain@umt.edu.pk; 2 Department of Anthropology and Sociology, Faculty of Social Sciences and Humanities, Universiti Kebangsaan Malaysia, Bangi 43600, Malaysia; vivienyew@ukm.edu.my; 3 Azman Hashim International Business School, Universiti Teknologi Malaysia, Skudai 81310, Malaysia; sehrish.malik@hotmail.com

**Keywords:** help-seeking behavior, Afghan refugee, scavenger, health care utilization, qualitative research

## Abstract

Scavengers are prone to various health problems, hence seeking healthcare is inevitable. Despite the importance of the help-seeking behavior of scavengers, it has not been addressed explicitly in the existing literature. Addressing this gap, this study intends to explore the help-seeking behavior of scavengers and to identify the factors that influence healthcare utilization among them. This qualitative study conducted thirty-one in-depth interviews through a semi-structured interview guide and analyzed them using thematic analysis. Andersen’s Behavioral Model of Health Service Use was employed to explore the findings. The findings showed that the scavengers utilized multiple healthcare options depending on the severity and reoccurrence of the illness. The process of help-seeking and health services utilization was largely influenced by the cost of the health service, long distance to the health facility, traveling cost and waiting time. The study highlights the need for scavengers’ enrolment in micro health insurance schemes. The initiative would facilitate scavengers’ access to medical care. Health awareness campaigns and the provision of free mobile medical services, especially at the landfill sites, would also improve curative treatment among scavengers.

## 1. Introduction

Scavenging, as in the extraction of useful items from solid wastes, is a popular activity among the urban poor [1]. Despite being an income-generating activity, scavenging is a hazardous activity that poses serious health risks [2]. Strenuous activities at the workplace, such as bending, heavy lifting, pulling, pushing, and carrying, as well as exposure to excrement, toxic substances, chemicals, sharp objects, and medical waste, leave adverse effects on both the physical and mental health of scavengers [3,4]. Potential health risks among waste workers include accidents, infections, musculoskeletal problems, gastrointestinal and respiratory disorders, and allergies [1,2,5,6,7].

The phenomenon of scavengers’ health is a well-explored topic. Studies conducted in different parts of the world such as Ethiopia [8], Ghana [9], India [6], Latin America [10], Malaysia [5], Nigeria [11], Pakistan [12] and Thailand [13] have given substantial evidence to believe that dealing with waste jeopardizes workers’ health. Although health problems are common among scavengers, little is known about their help-seeking behavior.

Help-seeking behavior is a multistage process that starts when a person perceives themselves as sick and shows a willingness to restore their health by taking help from a third party [14]. The process involves symptom perception, interpretation, evaluation, decision making and motivation to visit a health care professional for help [15].

Scavengers have not been studied frequently in the context of help-seeking; however, the existing knowledge indicates that scavengers usually self-medicate or use public health facilities [13,16,17]. Contrary to that, studies conducted on Indian scavengers [18,19] reported that the scavengers have a higher tendency of consulting private general practitioners. Besides that, a study conducted on e-waste workers [20] showed the utility of multiple health care options including self-medication, drugs from retail stores, traditional medicine, formal health care hospitals as well as clinics. The choice of health care was influenced by the severity of the disease, perceived benefit of the treatment, interpersonal interaction with the care providers, accessibility and quality of the service. Furthermore, Sustainable Development Policy Institute [21] noted that scavengers consider minor injuries and ailments as a routine matter and hardly consult the doctors until the symptoms get worse. Moreover, financial constraints also play a pivotal role in delayed treatment. Similarly, a study conducted on Pakistan scavengers [22] concluded that self-perceived low severity of health problems, longer waiting time, discriminatory treatment at the hospital, negative attitude of paramedical staff and inferiority complex are the main factors that contribute to delayed healthcare among scavengers. 

The Islamic Republic of Pakistan is situated in South Asia adjoining Central Asia and the Middle East. It is world’s fifth most populous county with more than 220 million residents [23]. In Pakistan, Afghan refugees dominate the scavenging activities [12]. The protracted history of Afghan mass migration to Pakistan started when the Soviet Union invaded Afghanistan in 1979. Over the years, millions of Afghans took refuge in Pakistan and approximately 1.4 million registered Afghan refugees are still residing in Pakistan [24]. Afghans relied on agriculture to make a living before fleeing [25,26]. Despite their agricultural expertise, they were neither provided with land for cultivation nor allowed to own it during their exile in Pakistan [27]. The affluent among them quickly established themselves in trade and business across Pakistan [28] whereas the rest, being uneducated, unskilled and relatively poorer than the native Pakistanis, took low-status jobs. Most of them chose scavenging as a sole survival strategy since it requires no special skills, training or resources to get started.

Sociologists have proposed different theories and models to predict human help-seeking behaviors, one of which is Andersen’s Behavioral Model of Health Service Use (ABM). The model provides a theoretical understanding of an individual’s decision to use health care services. The model argues that an individual’s tendency to use medical care is influenced by three key elements: (i) predisposing; (ii) enabling; and (iii) need factors. Predisposing factors such as demographic characteristics, social structural variables, and health beliefs influence one’s attitude towards health utilization [29]. Enabling factors, such as income, health insurance and the price of health services expedite or hinder the use of health services. Need factors include perceived need or evaluated need; perceived need refers to how people view their health and experience the severity of the symptoms whereas evaluated need refers to the professional medical measurement of health status and the need for health services [29].

Since the help-seeking behavior of scavengers has largely been overlooked in the available literature [30], the current study aimed to describe the scavengers’ help-seeking behavior and identify factors affecting health service utilization among the scavengers by using Andersen’s ABM.

It is believed that this study would contribute new knowledge on an under-researched area that would be useful in designing policies and plans concerning scavengers’ health care needs.

## 2. Materials and Methods

### 2.1. Study Design and Setting

This study used qualitative descriptive design, which aims to provide comprehensive summarization of the events experienced by individuals and groups in a simpler manner [31]. It is thought to be appropriate for providing simple descriptions of experiences and perceptions [32], especially in under-researched phenomena.

This study was conducted in the city of Gujrat, in the province of Punjab, Pakistan. As an industrial city, Gujrat generates a huge amount of solid waste that has consequently accelerated Afghan refugees’ involvement in scavenging activities. The city was considered an appropriate research site as it had settled Afghan refugee communities, enabling access to participants who met the inclusion criteria. Moreover, the researchers’ familiarity with the city’s language and dialects made rapport building, participants’ recruitment, and data collection easier. 

### 2.2. Participants

Scavengers in Pakistan can broadly be divided into two groups: native scavengers and Afghan refugee scavengers. Gypsy communities constitute the majority of the native scavenging population in the country. They could be defined as a Muslim, nomadic, socially and politically marginalized community that has been living in the country even before the partition in 1947. On the other hand, Afghan scavengers are the people who fled Afghanistan and have been living in Pakistan for decades. The phenomenon of scavenging in the country is widely attached to these refugees [33]. Illiteracy and socio-economic marginalization keep them far away from the formal job sector and leave them with no option but low-skilled and hazardous jobs to meet their ends [34]. For this research, Afghan scavengers were preferred over native scavengers for primarily two reasons: (i) Afghans constitute the majority of the scavenging population in the town; (ii) Afghans, unlike native scavengers, worked full-time. Therefore, they were more exposed to occupational risks and health impairments as full-time scavengers since they worked longer hours, on average 8–10 h daily.

To get the most relevant data a combination of purposive and snowball sampling was employed to access the individual who had experienced the phenomenon to be researched [35]. The blend of sampling helped identify a purposive and homogenous sample. Keeping in view the research objectives, research design, prior literature and current situation of the research site, the following criteria were set to select the participants:

1. Afghan refugees;
2. Adult male (as Afghan women are not allowed to work outside the home and children might not be able to share the true essence of their experiences);
3. Full-time scavenger;
4. Five-year work experience or above;
5. Willingness to share experiences.

### 2.3. Participant Recruitment

Despite being a native resident of Gujrat, the primary research found it difficult to gain access to and recruit the appropriate participants at the research site Afghan scavengers worked independently therefore, no formal or informal head regulated their activities or the ‘gatekeeper’ who could have ensured access to them. Consequently, locating informants on our own was difficult. After several unsuccessful attempts to persuade the potential participants both independently and with the help of Afghans, an Afghan agreed to introduce one of his willing relative scavengers to take part in the research. Thenceforth, with his assistance and snowball sampling technique, the sample started getting bigger. The process of the actual recruitment began with approaching and informally talking to the Afghan scavengers who matched the inclusion criteria. During such casual meetings, the purpose of the research, the importance of Afghans’ involvement and confidentiality of their information were conveyed. Upon potential informants’ consent to take part in the research, the convenient time and place of their availability were finalized. The demographics of the recruited participants are demonstrated in Table 1.

### 2.4. Sample Size

We used data saturation as a criterion to determine the sale size. Saturation was ascertained by keeping a record of when the codes first appeared and last appeared in the interviews [36]. The data collection was discontinued after 31 in-depth interviews when no new information was obtained from new participants [37].

### 2.5. Data Collection

To gather the lived experiences of the Afghan scavengers, this study predominantly relied on open-ended, face-to-face semi-structured, in-depth interviews to describe the health seeking behavior of the Afghan scavengers. A semi-structured interview guide was designed with the help of an intensive review of the prior literature. Five pilot interviews were conducted to assess the efficacy and limitations of the interview guide. The pre-testing revealed that scavengers, being uneducated, found few questions difficult to understand and subsequently were unable to share their experiences on it. Based on the feedback received from the scavengers, a revision was carried out by breaking down the complicated questions into simpler ones. It eventually improved the structure and the content of the interview guide [38]. The final list of well-planned, open-ended and pretested questions, including probes, was beneficial for this study as it increased the interviewer’s confidence level, made her more focused towards interviewees’ responses and less preoccupied with what to ask next and how to ask it [39]. Additionally, the open-ended nature of the questions encouraged the informants to discuss their experiences effortlessly and express their views openly. Before proceeding to the actual interview, as a prerequisite, the participants’ consent of engaging in the study was taken. The participants were hesitant to sign any document, and therefore, their verbal consent was recorded to ensure their voluntary participation. 

The primary researcher conducted all the interviews each lasting between 45–76 min depending on the informants’ ability to focus. Each interview commenced with introductory questions, primarily on the demographic information leading to the exploration of participants’ experiences and insights on their help-seeking behavior. Urdu, as the only shared language among the primary researcher and the interviewees, was used to obtain the data. The interviews were audio-recorded on a smartphone and then transcribed and translated into English. 

In the first phase, the lead researcher, being the “insider” and bilingual, translated all the interviews from Urdu to English before the analysis. In the next phase, a translator, familiar with the language and culture of the participants, was engaged to ensure the linguistic validity. Translating qualitative data from source language (Urdu) to target language (English) was challenging. At some points the lead researcher faced difficulties in conveying native meanings into English. Such situations were overcome by holding discussions with the research team and coming to an agreement.

### 2.6. Data Analysis

**Figure 1:**
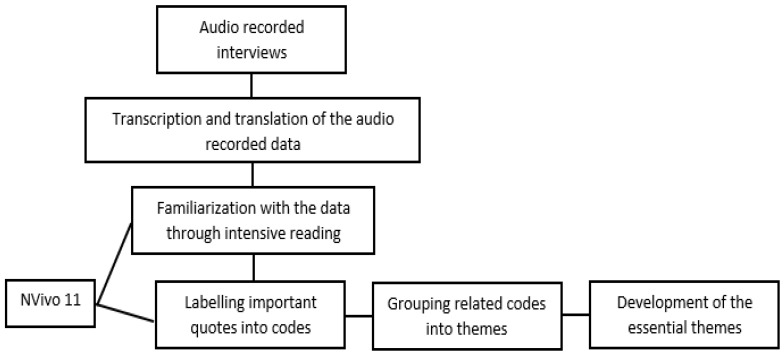
Process of data analysis.

The lead researcher (BM) used thematic analysis to analyze the transcribed data. The process started with exhaustive reading and re-reading of the transcripts to get familiar with the data (Figure 1). Then, all the recurrent ideas, words and phrases revealing the thematic aspects of the phenomenon and answering the research questions were highlighted and coded. A computer-assisted qualitative data analysis software (CAQDAS), NVivo 11 (QSR international, Melbourne, Australia), was used to facilitate the process of coding.

In the next step, all the codes were scrutinized to find connections between them and determine whether they can be grouped into themes. In the last step, superordinate or essential themes were developed by comparing and contrasting the previously identified themes. These themes were then shared with the co-researchers, and any disagreements were discussed until a consensus was established. Eventually, the essential themes summarized “what” the individuals have experienced in the phenomenon and “how” they have experienced it [40].

## 3. Results

A total of thirty-one Afghan scavengers, who matched the inclusion criteria, were interviewed over five months. Six themes, namely: (i) delay in seeking help; (ii) self-medication; (iii) visiting small private clinics; (iv) public hospitals; (v) private specialized hospitals; (vi) home remedies, traditional and spiritual healing, emerged from the data analysis. Table 2 summarizes the health problems faced by the informants and the treatment options they opt for. The subsequent sections describe the major findings.

### 3.1. Health and Illness among Scavengers

Health and illness are multidimensional concepts that have always been perceived distinctly by different communities. People’s socioeconomic level, occupation, and cultural views affect the way they conceptualize their illness. The Afghan scavengers in this study shared a common perspective of health. Unlike the traditional notion of health as an absence of disease, they described it as the ability to perform societal duties and obligations, particularly those linked to work.

Participant 11 (thirty-two-year-old): There’s always something wrong with us (scavengers). But these little issues (health problems) cannot prevent us from living our lives. As long as I can get to work every morning, I consider myself fit.

Participant 7 (twenty-four-year-old): Every night, I return home with some form of issue (health problem). It is part of my job now. But thank God I’m off to work the next morning, this is health and greatest blessing for me.

As far as the illness is concerned, majority of the informants considered it the outcome of their profession whereas a handful of them conceptualized it as inevitable and predestined. Participant 8 perceived disease as an unavoidable fate that must occur regardless of how hard a person tries to avoid it. Squalor and filth, he argued, could not cause a health problem if it is not in the fate, and personal protective equipment (PPE) cannot protect one from it.

Participant 8 (forty-year-old): …when there comes disease from the God then nobody can protect you from it. These theories that germs create diseases is a western propaganda, that’s meant for terrorizing people from developing countries. These theories are not true.

### 3.2. Making Healthcare Decisions

The decision to seek treatment is influenced by various personal and social factors, and especially by how someone defines illness. The Afghan scavengers delayed their health seeking as they considered illness as an inability to carry out work-related activities and themselves as brave enough to ignore the mild symptoms.

Participant 4 (twenty-two-year-old): …I being the pathan (Afghani) bear the joint pains. I only visit a doctor when the pain is out of control.

The process of health-seeking decisions starts when the illness disrupts the day-to-day activities. Gender plays an important role in deciding when to seek medical care. In Afghans patriarchal household men dominate the decision making. Being male, the participants of the study were independent to make health care decisions on their own. Most importantly, the head of the family were free to choose the right health care options for themselves and their family members. Whereas, the young, unmarried men, made the decisions in consultation with their families.

Participant 21 (nineteen-year-old): I was experiencing backache for few days now. I drank green tea to get relief, but it didn’t change much. Then my father gave me money and asked to visit the doctor.

Advice from male Afghan relatives and friends, living in Pakistan, were sought while interpreting symptoms and deciding on treatment choices for chronic illnesses. These lay referral networks could encourage or discourage professional consultation.

Participant 8 (forty-year-old): I discuss my issues with my paternal cousin. I told him about my stomach-aches the other day. He said the symptoms are not worrisome because he has had identical ones before, and they went away on their own. So, I didn’t consult the specialist doctor for that.

### 3.3. Delay in Seeking Behavior

A low rate of seeking medical help in the symptoms experiencing stage was observed among Afghan scavengers. Instead of early detection and treatment, they considered symptoms as insignificant and left them unattended to heal themselves. Remedial actions were taken when the symptoms fully developed and hindered day-to-day activities. Delay in seeking medical advice had serious repercussions for many:

Participant 21 (nineteen-year-old): …at that time, we (the family) delayed the treatment as we did not have enough money. When I was close to death then I was taken to a hospital. My family was sure that I will not get cured so they cried for me. I was taken to a private hospital as the DHQ (District Headquarter Hospital) refused to admit me because of my serious condition.

Participant 9 (twenty-six-year-old): I consult a doctor for almost every sort of illness, but before visiting the doctor I wait for a while hoping for my body to heal itself.

### 3.4. Self-Medication

Self-medication, a way of treating self-diagnosed symptoms without seeking help from medical professionals, was widely practiced by the Afghan scavengers. Injuries and aches, as the most common health problem, were responded to with self-medication the most. After the initial response to injuries at the workplace (wrapping a piece of cloth or polyethylene bag, sprinkling clay or pouring petrol on the wound), it is cleaned with plain water and treated with ointment.

Participant 6 (forty-five-year-old): Once I am back (from work), I clean the wound thoroughly and apply any ointment available at home. I have one blue-colored ointment which I bought from the store (pharmacy), but if my cut is minor, I then just maintain the cleanliness and let it heal itself.

Apart from everyday minor injuries, several respiratory and gastrointestinal illnesses including the common cold, sore throat, abdominal pain and indigestion were also initially treated with the help of over-the-counter drugs.

Participant 10 (twenty-eight-year-old): I hardly visit a doctor for that (lower back pain). Usually, I get the bandage from the store (pharmacy) and wrap it around the affected area, and it feels better afterward.

### 3.5. Visiting Small Private Clinics

Private healthcare was the most prominent service utilized by the Afghan scavengers due to its responsiveness and low-cost treatment within the proximity. In these clinics, the consultation was free, and the patients were charged only for the drugs.

Participant 11 (thirty-two-year-old): If my family members or I do not get relief from the medicine bought from the store, then we visit the doctor.

The doctor charges PKR (1 USD is equal to 158.48 Pakistani rupees (PKR) according to the currency exchange rate as at 24 June 2021) 20 (USD 0.13) for medicine whereas consultation is free of cost. The medicine usually consists of one injection and a few syrups.

Repeated visits had allowed the participants to develop a bond with the doctors over time. The frequent nature of the doctor–patient interaction further helped scavengers to avail special discounts and relaxation is fee payment at their convenience:

Participant 17 (forty-six-year-old): Sometimes I tell the doctor that I’m a poor person, so he gives me discounts. He also charges less fee from me than that of native residents. A few days back when my wife was not feeling well, we went to see the doctor and I told him that I’m not having enough money. His fee was PKR 200 (USD 1.26), but he asked for PKR 100 (USD 0.63) after the discount.

Participant 20: (thirty-nine-year-old): The other day my doctor from the nearby clinic met me in the masjid and inquired me about my stomachache. On my last visit to the clinic, he gave me a discount as well.

### 3.6. Public Hospitals

The public hospitals were found out to be one of the least preferred help-seeking options among the Afghan scavengers. Distance to the public health facility was cited as one of the most important barriers hindering the utilization of specialized health services. Moreover, the absence of personal conveyance and hefty transport fares limited the participants’ access to public hospitals only for serious health issues:

Participant 22 (thirsty-five-year-old). The government hospital is very far from us. The private doctor charges PKR 20 (USD 0.13) for consultation and medicine whereas only getting to DHQ (public hospital) needs PKR 60 (USD 0.38) as transport fare. So, we only go there once there is a serious illness.

The long waiting time to see a physician was also discussed as a hindrance. Specialized and free treatment attracts a large number of patients to visit public hospitals making it an overcrowded place. Prolonged waiting hours dissuaded scavengers to public health facilities:

Participant 11 (thirty-two-year-old): The treatment in government hospitals is relatively cheaper, but we can’t afford to go there as it is far away from our area and needs familiarity with the officials to get your work done on time. Apart from this, the Gujrat DHQ is a very busy place. One morning I went there to visit a doctor in the hospital, but my turn did not come until evening as there was a rush and people were pushing and pulling each other.

Apart from distance and long waiting time, personal reference was also discussed as an obstacle. Participants were of the view that while waiting in the queue, people with personal references, either with the gatekeeper or the doctor himself, get to see the physician at the earliest. It subsequently increases the wait time for a patient with no reference like them:

Participant 12 (thirty-seven-year-old): I do not go to the government hospitals as there; personal references are needed to get treatment. That’s why I consult nearby private clinics.

Participant 28 (twenty-five-year-old): We rarely visit the government hospital since the staff there prefers the natives and do not attend us well. They make us wait a little longer. We only go to the public hospital when the babies need to be vaccinated.

### 3.7. Private Specialized Hospitals

In the process of seeking professional medical care, the specialized private hospital were least utilized by the scavengers. Being expensive, these hospitals were approached as the last option to treat the illness.

Participant 27 (forty-year-old): I visited the doctor and complained about a headache, so he asked me to go for the X-ray which cost PKR 4000 (USD 25). After that, he told me I have a problem with my brain and advised me to continue medicine which I could not.

Although the persistent symptoms made the scavengers visit the high-end hospitals, their medication compliance was largely affected by the expensive treatment. Moreover, repeated visits to the hospital and transportation fares all contributed to the non-adherence of the treatment.

Participant 27 (forty-year-old): I have recently stopped using the medicines as I can’t afford medicine and the doctor’s fee. My wife asked me to visit the doctor yesterday, but I did not go as I do not have enough money for that.

### 3.8. Home Remedies, Traditional and Spiritual Healing

As compared to biomedicines, complementary and alternative medicines were the least popular. Home remedies were common among all. The most conventional remedy, to treat aches and upper respiratory problems, was taking green tea. The participants regarded green tea as a means of warmth and relaxation that provides relief in fatigue, body aches, common cold and sore throat:

Participant 4 (twenty-two-year-old): I drink a cup or two of green tea when I get too tired. It helps me become relaxed. We cannot afford to buy milk as it’s too expensive. Milk is PKR 100 (USD 0.63) per liter, hence green tea is an alternative to regular milk tea for us.

Unlike home remedies, spiritual healing was used by Afghan scavengers either as a method to complement biomedicine or after getting disappointing from conventional medicines. The healing procedure, locally known as damm, involved the recitation of the Quranic verses or authentic prayers by a cleric that was then blown on the afflicted area in the hope to get it cured.

Participant 15 (twenty-five years old): At first, my family and I consult a doctor for all kinds of diseases but if it could not cure the problem then we visit the maulana for damm. It is usually for a disease that is a serious one and the doctor cannot treat it well.

The traditional way of healing was only reported for sore throat. Although the method was widely used to treat children, few participants admitted using the method especially when they were short of money. The practice, locally known as Talu, was a technique to massage and soften the swollen veins on the hard palate, believed to be the reason for sore throat.

Participant 7 (twenty-four-year-old): She (healer) simply takes ash from the stove and massages it to the hard palate of the patient. It softens the affected veins and makes the patient feel perfectly well afterward. Not only I but all the Afghans around me use this technique for tonsillitis and sore throat.

## 4. Discussion

This study aimed to describe how scavengers perceive their illness and who they consult for treatment. This is one of the few studies that have explored the help-seeking behavior of scavengers in detail. Andersen’s Behavioral Model of Health Service Use (ABM) [29] argues that need factors such as a patient’s subjective interpretation of somatic symptoms determine whether the person seeks medical help right away or delays it. A person is therefore more likely to seek medical help if they consider their illness severe and desire it to be eliminated. Likewise, Afghan scavengers’ decision to seek medical help was highly influenced by how they perceived and interpreted their symptoms. Unlike the conventional perception of health as the absence of disease, they characterized health as the ability to carry out daily tasks, particularly those related to their jobs. Health, according to scavengers, was defined as the ability to perform social roles and the absence of hindrance in doing so. As a result, informants did not consider a health problem until it began to interfere with their day-to-day responsibilities. The findings of this study revealed that the scavengers perceived mild physical pain, cuts, and discomforts as not requiring immediate attention. As a result, the remedial help was delayed until the symptoms had fully developed and interrupted daily activities. This study explains that low income and self-perceived low severity of health problems were key factors in treatment delays, which is in accordance with previous research in Pakistan [21,22].

Afghan scavengers’ decision to engage in health behavior was influenced by various personal and social factors, most importantly how they define their symptoms, their position in the family and their lay referral networks. Response to pain and distress is influenced by culture and ethnicity. The reaction to pain varies and reflects the beliefs of the group [41]. Among Afghans, men are considered strong and reluctant to express their pain and distress. Therefore, the participants considered themselves strong enough to overlook minor illnesses and allow them to heal on their own.

The majority of the participants, being male and family heads, evaluated their symptoms and made their own healthcare decisions, whereas the young and unmarried scavengers, being dependent on the family, relied on their family to interpret their symptoms and prescribe treatment. In the case of chronic illness, however, the lay referral networks, which were made up of Afghan men, assisted the participants in validating the nature of illness and deciding on a course of action [42].

When seeking help became unavoidable, scavengers responded to their illness differently, depending on the severity and reoccurrence of the symptoms, the cost of the treatment and the distance to the nearest health facility. Self-medication was reported to be one of the most prominent treatment options among the scavengers [16]. Initially, all the health problems ranging from minor injuries to major illnesses were treated with over-the-counter medication. Easy access to medicines without doctor’s consultation, persistent illness, large family size, long working hours and financial constraints all contributed to the popularity of self-medication. This finding is also consistent with the research conducted in Ghana [20] and Vietnam [17] where scavengers chose to self-treat with over-the-counter drugs. In line with the ABM, the predisposing variables such as demographic characteristics and social structural variables influence healthcare utilization among people. Similarly, in this study, it was found that large family size, illiteracy, ethnicity and health beliefs were found to cause a delay in health service utilization among the scavengers. Participants believed that minor diseases pose no serious threats to their health, so they did not need medical care. Furthermore, they were proud of their ethnicity and considered themselves to be strong enough to prevent minor ailments.

Private healthcare facilities came into play when an illness took longer than usual to cure with self-medication. Due to their cost-effective healthcare delivery, participants preferred small private clinics located inside the scavengers’ communities over public healthcare facilities. Private clinics, such as those reported by Nam and colleagues [17] were also popular among the participants. These healthcare centers were easily accessible by trekking or riding a bicycle as they were located in a residential area. Apart from their accessibility, these clinics’ long operating hours were found to be an essential factor. In line with the ABM, enabling factors like low household income, affordable treatment, geographic accessibility, flexible working hours increased scavengers’ utilization of private health facilities.

Scavengers were able to avoid skipping work and waiting in large lines at government hospitals since private practitioners worked until late at night. Scavengers who visited these clinics on a regular basis also developed a bond with the doctors, which resulted in special discounts and the ability to pay bills in installments. The unusual doctor–patient social bond was possible as these doctors lived and worked in close proximity to Afghan refugee settlements, allowing them to engage regularly at masjids and grocery stores. Their acquaintance further made the doctors familiar with scavengers’ economic conditions and favored them with consultation fee discounts. This finding of the study corresponds with the findings of the studies conducted on Indian scavengers [18,19] that documented private healthcare as the most preferred choice among scavengers due to its convenience and less time-consuming nature.

Despite the availability of specialist doctors, nominal consultation fees and free medications, the public hospitals were observed to be one of the least preferred help-seeking options among the scavengers. The distance to the health facility was a key barrier to public healthcare utilization. Most of the participants lived on the outskirts of the city far away from public hospitals, and therefore getting to a public health facility was difficult. Moreover, the absence of personal and public transport exacerbated the situation. Apart from distance to the healthcare facility, long waiting time to see a physician in these hospitals was reported as a hindrance. Scavengers considered it a major obstacle as it made them skip work and wait for their turn. Moreover, a few participants claimed to face discrimination at the public hospitals and to have unequal access to medical care.

When all the aforementioned methods failed to eliminate the symptoms, specialized private hospitals were explored in the process of obtaining medical advice. At this stage, the disease had fully developed, was severe, and recurrent. These hospitals, unlike public health facilities, were timesaving but expensive, hence only a few participants visited them. In line with the ABM, need factors such as persistence and severity of symptoms, absence of relief, hindrance in day-to-day activities and desire to relinquish the sick role all increased the odds of specialized service uptake. However, pricy treatment in these hospitals made scavengers switch back to small private clinics.

A study conducted by Asampong and colleagues [20] reported the popularity of traditional medicines among scavengers. Contrary to that, the participants rarely used complementary and alternative medicines. Home remedies were common among scavengers, but they were only used as an initial response to the illness. A few participants also claimed to use spiritual healing to complement conventional medicines. Despite the prevalence of complementary and alternative medicines, scavengers predominantly preferred biomedicines as they provided instant relief and prevented days off from work.

The Afghan scavengers were largely interested in symptomatic treatment, aiming to reduce or eliminate symptoms that interfere with a person’s ability to function and their quality of life. Small private clinics in the research site offered treatments that temporarily reduced the severity of ailments while ignoring the underlying causes of the illness. Consequently, the participants stopped taking the medications as the intensity of the disease diminished, making the symptoms recurring. Symptoms’ reappearing indicates that the disease is still active in the body, although having been suppressed previously.

Despite contradicting evidence on the efficacy of ABM [43,44], our findings show that it is a useful framework for understanding the Afghan scavengers’ decision to use health care services in Pakistan. The model suggests that that a person’s willingness to utilize a service is determined by three factors: predisposing components; enabling components; and need components. Predisposing factors indicate a person’s tendency to utilize health services. Healthcare utilization among the participants was influenced by predisposing factors such as big family size, illiteracy, and ethnicity. Scavengers’ general knowledge and awareness of health risks associated with waste handling, and living in an urban area both enabled them to visit the doctors frequently. However, their beliefs that minor diseases pose no serious threats to their health and therefore they requires no medical care, and that their ethnicity is strong enough to prevent minor ailments, delayed treatment seeking among them. Enabling factors, on the other hand, refer to resources that could facilitate or impede access to services. Among scavengers, low household income, affordable treatment, geographic accessibility, and flexible working hours increased scavengers’ utilization of private health facilities. Conversely, large family size and the absence of health insurance normally hindered their access to specialized hospitals and compliance with curative treatments. Need factors, as an individual’s perceived need or medical professional’s assessment of health status and the need for health services, also influenced scavenger’s healthcare service utilization. Individual need factors such as persistence and severity of symptoms, absence of relief, hindrance in day-to-day activities and desire to relinquish the sick role as well as doctors’ advice for medical screening and curative treatment all encouraged scavengers to seek private specialized health services. Our research data does not support new constructs in the model.

This study highlights an urgent need of communicating the importance of curative treatment to the scavengers through health awareness initiatives. The knowledge of how to maintain, restore, and promote health will go a long way. The scavengers’ low educational level, on the other hand, limits the contemporary usage of powerful social media platforms for awareness campaigns [45]. Therefore, the appropriate strategies may include the dissemination of care-seeking and prevention information at dumpsites through loudspeakers, participation of local religious leaders, influential Afghan refugees and scavengers, as well as experts in health promotion programs, employment of mobile network announcement at the scavenging communities and provision of free mobile medical services, particularly at the landfill sites. Moreover Afghans and especially scavengers’ enrolment in on-going national micro health insurance scheme, the *Sehat Sahulat* Program, that aims to provide free access to health services in both public and private hospitals for families living below the poverty line [46], would eliminate health care disparities and ensure equal access to health facilities. Apart from that, the allocation of free and equal public sector healthcare facilities requires policymakers’ attention.

Although this study provides a comprehensive description of the help-seeking behavior of scavengers in Pakistan, it also has some limitations. The current study used a small, homogeneous group of adult male scavengers, so it is unlikely to represent the experiences of people of other ages. Another disadvantage of this study is the inability to generalize the findings. This qualitative study was solely designed to provide rich description of the health seeking behavior of Afghan scavengers and not to draw broader conclusions. The study used a small sample of Afghan scavengers, therefore, no claim can be made that the findings are representative of all Afghan scavengers in Gujrat and the surrounding areas. However, the use of a mixed method approach in future studies can overcome this limitation.

## 5. Conclusions

Occupational illnesses are common among scavengers that need remedial help. Scavengers utilized different treatment options based on the nature of the health problem. Self-medication and over-the-counter drugs were the most prevalent treatment option for minor illnesses, whereas, the most persistent health problems were dealt with by using private healthcare facilities. These centers were largely known for quick and cheap treatment. Despite the highly subsidized but specialized treatment, public healthcare was still avoided due to long distances to the health centers, non-availability of transportation, hefty transport fares, longer waiting hours and discrimination at the hospitals. The findings show that health awareness, free mobile clinics and scavengers’ enrolment in health insurance programs can improve their access to quality healthcare.

## Figures and Tables

**Table 1:** Participants’ demographics.

Demographics	*n* = 31
**Age**	
Below 20	3
20–29	18
30–39	3
40–49	6
Above 50	1
**Marital Status**	
Married	25
Single	6
**Family structure**	
Nuclear	11
Joint	20
**Educational level**	
No schooling	23
Less than primary	6
Primary	2
**Place of birth**	
Pakistan	17
Afghanistan	14
**Total years of living in** **Pakistan**	
15–25	19
26–36	12
**Total years in the profession**	
6–12	13
13–19	9
20 and above	9
**Family income (USD)**	
Less than 100	6
100–199	17
200–299	6
**Total family members**	
Up-to 5	4
6–15	16
16–25	11
**Total dependents**	
1–10	20
11–20	10
21 and above	1

**Table 2:** Health problems and their treatments.

Categories	Health Problem	Total Participants Experienced the Health Problem	Common Treatment Options	Total People Used the Treatment	Factors Associated with Treatments Options
**Injuries and aches**			- Leaving the area unattended	15	- Perception about the illness - Perceived severity of the disease - Frequency of the symptoms - Availability of the treatment - Quick relief - Affordability of the treatment - Accessibility to the healthcare facility - Waiting time to see a care provider
	Lower Back pain	29	- Consulting clinics in the locality	14	
			- Visiting a drugstore for painkiller	7	
			- Wrapping bandage to keep the area warm	6	
	Multiple joint pains (elbow, wrist, knee, shoulder)	28	- Sleeping, taking rest and massaging the area with oil to relax the muscles	5	
			- Green tea for relief	5	
			- Consulting orthopedic	3	
			- Religious healers	2	
	Glass injuries	25	- Wrapping the wound with available cloth, polythene bag or paper	13	
			- Holding the affected area tightly with a hand	3	
			- Doctor consultation for deeper cuts	3	
			- Leaving small cuts unattended	2	
			- Buying bandages from a medical store	2	
			- Applying ointment available at home	1	
			- Sprinkle clay on the wound and some prefer to pour petrol on the bleeding	1	
**Allergies and infections**	Eye allergy/irritation	18	- Washing eye with fresh water	10	
			- Visiting drug store	7	
			- Visiting nearby clinics	6	
	Skin allergy	17	- Consulting skin and eye specialist at DHQ hospital	5	
			- Consulting skin specialist in a private hospital	4	
			- No treatment	3	
	Dog bite	7	- Applying oil and red chili paste on the bite	2	
			- Consulting nearby clinic	2	
			- Visiting DHQ	1	
			- No treatment	1	
			- Religious healing	1	
	Typhoid	9	- Consulting nearby doctor	9	
			- Visiting a private hospital	2	
			- No treatment	1	
	Hepatitis	4	- Religious healing	1	
**Respiratory and gastrointestinal disorders**			- Taking chicken soup or green tea	7	
			- Consulting nearby clinics	7	
			- Visiting drug stores	5	
	Common cold	27	- No treatment	4	
			- *Joshanda* (herbal tea)	2	
			- Green tea with black pepper powder	1	
			- Green tea with lemon juice	1	
	Poison from gaseous pollution	21	- Leaving the workplace immediately	10	
			- Taking rest under the shade	6	
			- Drinking water to freshen up	3	
			- Consulting the nearby doctor	2	
	Sore throat	24	- Using chicken soup, green tea and *Joshanda*	9	
			- Drug store for syrup	6	
			- Folk way of healing	4	
			- No treatment	2	
			- Religious healing	2	
			- Visiting clinics in the locality	1	
	Chest infection	5	- Consulting nearby clinics	4	
			- Visiting nearby clinics	2	
	Pneumonia	3	- No treatment	1	
			- Medication from the drug store	1	
	Abdominal pain and impaired digestion	26	- Consulting nearby doctor	9	
			- Drugstore	7	
			- No treatment	4	
			- Visiting a specialized doctor	2	
			- Using carbonated drinks with or without herbal powder for digestion	2	
			- Religious healing	1	
			- Drinking goat milk right forms the teats	1	
	Food poisoning and diarrhea	22	- Seeing a nearby doctor	18	
			- Visiting DHQ hospital	5	
	Ulcer	5	- No treatment	3	
			- Visiting a private hospital	2	
	Cholera	2	- Religious healing	1	

## Data Availability

The data presented in this study are available on request from the corresponding author. The data are not publicly available due to ethical restrictions.
